# Comprehensive analysis to identify the neurotransmitter receptor-related genes as prognostic and therapeutic biomarkers in hepatocellular carcinoma

**DOI:** 10.3389/fcell.2022.887076

**Published:** 2022-08-05

**Authors:** Xiaoqiang Wang, Yiran Li, Yumiao Shi, Jiamei Luo, Yiqi Zhang, Zhiying Pan, Feixiang Wu, Jie Tian, Weifeng Yu

**Affiliations:** ^1^ Department of Anesthesiology, Renji Hospital, Shanghai Jiaotong University School of Medicine, Shanghai, China; ^2^ Department of Intensive Care Medicine, Eastern Hepatobiliary Surgery Hospital, The Third Affiliated Hospital of Naval Medical University, Shanghai, China

**Keywords:** neurotransmitter receptor, hepatocellular carcinoma, LASSO model, bioinformatic analysis, TCGA

## Abstract

**Background:** Hepatocellular carcinoma (HCC) is a highly heterogeneous disease with high morbidity and mortality, which accounts for the fourth most common cause of cancer-related deaths. Reports suggest that the neurotransmitter receptor-related genes (NRGs) may influence the tumor microenvironment and the prognosis of patients with HCC.

**Methods:** The clinical information and RNA-seq data of patients with HCC were acquired from the ICGC-LIRI-JP dataset and the TCGA-LIHC dataset. Effects of 115 NRGs on the prognosis of HCC patients were analyzed in the ICGC-LIRI-JP dataset. The least absolute shrinkage and selection operator (LASSO) regression model was utilized to generate a risk score formula based on the critical NRGs. Next, the risk score effectiveness was validated both in the TCGA-LIHC dataset and in our clinical HCC samples. Based on the risk scores, patients with HCC were divided into two groups. Moreover, differentially expressed genes (DEGs) were screened. The gene ontology (GO) was used to analyze the functional enrichments of DEGs and to identify potential signaling pathways. To test the diagnostic effectiveness of our model, the receiver operator characteristic curve (ROC) analysis and nomogram were used. Finally, potential targeted drug prediction was performed based on DEGs of nine clinical HCC samples.

**Results:** Nine NRGs were correlated significantly with the prognosis of patients with HCC, and eight NRGs were successfully included in the LASSO regression model. The Kaplan-Meier analysis of overall survival (OS) suggested that patients in the high-risk score group had worse prognosis; on the other hand, ROC analysis revealed a high prognostic value of the risk score in HCC. Several critical signaling pathways, such as lipid metabolism, organic acid metabolism, cell migration, cell adhesion, and immune response, were enriched both in public datasets and clinical samples. Nomogram results also suggested that the risk scores correlated well with the patients’ prognosis. Potential targeted drugs prediction revealed that tubulin inhibitors might be the promising drugs for patients with HCC who have high risk scores based on the NRGs.

**Conclusion:** We established a prognostic model based on critical NRGs. NRGs show a promising prognostic prediction value in HCC and are potential therapeutic targets for the disease treatment.

## Introduction

Cancer is still the leading cause of death worldwide (Bray et al., 2021). The Global Cancer Incidence, Mortality, and Prevalence (GLOBOCAN) 2020 estimated that there were 19,292,789 cancer cases and 9,958,133 cancer deaths in 2020 globally (Sung et al., 2021; Xia et al., 2022). Hepatocellular carcinoma (HCC) is one of the most common cancers worldwide among all types of cancers and is especially prevalent in Southeast Asia (Llovet et al., 2016; European Association for the Study of the Liver, 2018). According to related reports (Xiao et al., 2019; Sarin et al., 2020; Xia et al., 2022), HCC is the third and second leading cause of death among all cancers in the world and China, respectively. In 2020, China contributed more than 45% of new cases and deaths from HCC. Additionally, the high heterogeneity and lack of biomarkers for prognosis prediction make it more challengeable for a HCC treatment (Qing et al., 2021).

To date, the main treatments considered for HCC are surgical resection, liver transplantation, and local ablation. However, the HCC recurrence and metastasis rate after treatments remains high, which greatly affects the prognosis of patients with HCC (2018). Studies have shown that the five-year survival for localized HCC is about 32%, while it drops drastically to 2% for patients with distant metastasis (Siegel et al., 2022). As a result, high recurrence rate and low survival rate remain a major unmet medical need. New targets for HCC individual therapy and early warning are of high significance and clinical value for improving outcomes.

Neurotransmitters are chemicals that help signals travel from one cell to another, which plays a key role in tumor microenvironment ecology. According to different chemical structures and properties, neurotransmitters are separated into four major types as follows: 1) acetylcholine (ACh); 2) amino acids, including glutamate, glycine, and gamma-aminobutyric acid (GABA); 3) biogenic amines, that consists of dopamine, norepinephrine (NE), epinephrine (E), and serotonin; and 4) neuropeptides, including but not limited to neuropeptide Y (NPY), neurotensin, and many others (Jiang et al., 2020a). As important neural signaling messengers, studies have suggested that neurotransmitters and their receptors contribute to tumor proliferation, tumor angiogenesis, and tumor metastasis (Boilly et al., 2017; Monje et al., 2020) through multiple mechanisms. Neurotransmitter receptors are varied and mainly consist of eight categories as follows: glutamatergic receptors, glycinergic receptors, dopaminergic receptors, histaminergic receptors, adrenergic receptors, 5-HT receptors, GABAergic receptors, and acetylcholinergic receptors. Cancer cells could not only produce and secrete neurotransmitters but also neurotransmitter receptors that are widely expressed in cancer cells (Jiang et al., 2020a; Zahalka and Frenette, 2020). Additionally, neurotransmitter receptors are also widely expressed on the immune cells’ surface and are regulated by their corresponding neurotransmitters, thus affecting tumor immune responses (Jiang et al., 2020b; Cervantes-Villagrana et al., 2020).

A complex role of neurotransmitter receptors in HCC has been indicated in the literature. Take the dopamine receptor as an example, Zhang et al. (2016) reported that moderate intensity swimming that produces dopamine significantly inhibits the HCC cells’ invasion both *in vitro* and *in vivo*. However, other studies have shown that patients with high dopamine receptor 1 (DRD1) expression have a worse prognosis, while dopamine and its receptor can promote tumor progression by PI3K/AKT signaling pathway activation (Yan et al., 2020). In addition, an increasing number of studies have focused on exploring interactions between adrenergic receptors and HCC progression (Wu et al., 2016; Hong et al., 2021). A study by Liu et al. (2021) showed that environmental stress could induce anti-tumor immunity and could sensitize immunotherapy against HCC by modulating β-adrenergic receptors/CCL2 axis. Nevertheless, Wu et al. reported that β2-adrenergic receptors promoted HCC progression and sorafenib resistance by inhibiting the autophagic degradation of hypoxia-inducible factor-1α (HIF-1α). Therefore, controversies remain, and more comprehensive analyses are needed.

Herein, we systematically analyzed the impact of 115 neurotransmitter receptor-related genes (NRGs) within eight categories through the two public HCC datasets and one clinical HCC cohort. Furthermore, we identified eight critical NRGs that significantly influence the overall survival (OS) of patients with HCC. We constructed a LASSO regression model to predict the prognosis of patients with HCC, utilizing the eight critical NRGs. The results demonstrated that NRG expression levels could effectively predict the HCC malignant degree through our model. Moreover, we also identified several potential signaling pathways that may be involved in HCC development. Together, this study suggests NRG dysfunction may be a potential target for HCC treatment.

## Methods

### Data acquisition from the ICGC-LIRI-JP dataset and the cancer genome atlas-liver hepatocellular carcinoma dataset

The total transcriptome sequencing (RNA-seq) data and clinical information of patients with HCC were acquired and downloaded from the International Cancer Genome Consortium (ICGC) portal (https://dcc.icgc.org/projects/LIRI-JP) and The Cancer Genome Atlas Liver Hepatocellular Carcinoma (TCGA-LIHC) dataset (https://tcga-data.nci.nih.gov/tcga/), respectively. Furthermore, the 115-NRGs list was obtained from the National Center for Biotechnology Information, United States National Library of Medicine (https://www.ncbi.nlm.nih.gov/gene/).

### Establishment of the prognostic model based on neurotransmitter receptor-related genes

The univariate cox analysis was used to screen the NRGs for prognostic value. The cut-off *p*-value was set at 0.05, and the selected survival-related genes were used for further study. Next, we utilized a LASSO regression model (Alhamzawi and Ali, 2018) for predicting the prognosis of patients with HCC, using the “glmnet” R package and integrating survival time, survival status, and gene expression data. Then, we used the 10-fold cross-validation method to obtain the most optimized model. Finally, eight critical NRGs were included in the LASSO regression model, and the minimum criteria determined the penalty parameter (λ). The risk score was calculated using the formula: risk score = (gene A expression × *a*) + (gene B expression × *b*) … + (gene N expression × *n*). Then, *a*, *b*, and *n* represented the regression coefficients. Based on the risk score median, patients with HCC were divided into the high-risk group and the low-risk group.

### Validation of the prognostic model based on neurotransmitter receptor-related genes

To validate the model’s effectiveness, the survival analysis between two risk groups was carried out using the “survminer” R package and the log-rank *t* test. Next, we used the “pROC” R package to analyze the time-dependent ROC based on survival time, survival status, and risk score of patients with HCC. In the meantime, we analyzed the relationship between different risk scores and follow-up time, the survival status, and the changes in eight critical gene expressions. The results were presented by using online bioinformatic analysis tools such as Sangerbox 3.0 (http://vip.sangerbox.com/home.html).

The ICGC-LIRI-JP dataset was regarded as the internal validation cohort, and the TCGA-LIHC dataset was regarded as the external validation cohort to validate the accuracy and availability of our model. Finally, a clinical trial was carried out in our center, and the obtained HCC samples (clinical cohort) were used for further validation.

### Principal component analysis and t-distributed stochastic neighbor embedding analysis

The PCA and t-SNE analysis were performed by using “Stats” and “Rtsne” R packages, respectively, to visualize the sample distribution between the high-risk group and the low-risk group. The results were then presented by using the “ggplot2” R package and the website iDEP.95 (http://bioinformatics.sdstate.edu/idep/) (Ge et al., 2018).

### Differentially expressed genes screening and distributions

The DEGs screening was performed by using the “limma” R package. Briefly, we first carried out a multiple linear regression by using the function “lmFit” and then computed the moderated t-statistics, moderated F-statistics, and log-odds of differential expression by empirical Bayes moderation of the standard errors towards a common value. The DEGs were screened by the threshold *p* value < 0.05, FDR < 0.05 and fold change > 1.5 for the ICGC-LIRI-JP and TCGA-LIHC datasets, while *p* value < 0.05 and fold change > 1.5 for the clinical cohort.

To visualize the distribution of DEGs, the map and clusters of DEGs were created using the dimension reduction algorithm t-SNE, and this was created by the website iDEP.95 (http://bioinformatics.sdstate.edu/idep/). In the meantime, the top 20 upregulated and downregulated DEGs heat maps were presented using the “ggplot2” R package.

### Functional enrichment analysis

To understand the potential mechanisms and signal pathways which DEGs participated in, the gene ontology (GO) enrichment analyses and circle plots were performed using the “clusterProfiler” R package (Mi et al., 2019). The minimum number and maximum number of genes in the cluster were 5 and 5,000, respectively. The significantly different GO terms and signal pathways were defined as the threshold *p* value < 0.05 and FDR < 0.1. The results were visualized by using the “ggplot2” R package.

### Constructing protein-protein interaction networks of differentially expressed genes

To understand the potential relationships of DEGs in protein level, the authors constructed a PPI network using the Search Tool for the Retrieval of Interacting Genes (STRING) online database (http://version10.string-db.org/) (Szklarczyk et al., 2015). The interactions with an interaction score <0.9 and the genes that had no direct/indirect interactions with ADRB2 would be hidden.

### Establishment of a prognostic nomogram for hepatocellular carcinoma

By integrating risk score, age, sex, race, and TNM stage into the Cox regression model, we evaluated the significance of these factors in predicting the OS of patients with HCC in the TCGA-LIHC dataset. Also, a novel prognostic nomogram was developed to offer a reliable and quantifiable method for predicting patients with HCC survival. The results were presented by using online bioinformatic analysis tools like Sangerbox 3.0 (http://vip.sangerbox.com/home.html).

### Potential targeted drugs prediction and interactions between chemicals and genes

The connectivity map database (CMAP; https://clue.io/, data version: 1.1.1.2) was used to explore the potential targeted drugs for HCC treatments, which had a high-risk score based on the prognostic model. As a collection of genome-wide transcriptional expression data from cultured human cells treated with bioactive small molecules, the CMAP database can assist researchers in discovering the functional connections between genes, drugs, and diseases through the transitory feature of common gene-expression changes. We could acquire the candidate drugs which resulted in opposite gene changes in HCC by inputting upregulated genes of DEGs and downregulated genes of DEGs into the CMAP. The top six potential drugs in HCC cell lines were listed (ranked by the correlation score).

To further explore the interactions between the candidate drugs and genes, the STITCH (version 5.0) database was used, and the correlated genes were shown in the network (http://stitch.embl.de/) (Szklarczyk et al., 2016).

### Hepatocellular carcinoma patients recruitment and clinical hepatocellular carcinoma samples collection

This was a prospective observational study. The study complied with the Helsinki Declaration and the Consolidated Standards of Reporting Trials (CONSORT) statement, and this was approved by the Renji Hospital Ethics Committee (KY2020-185). The written informed consents were obtained from all patients or authorized family members. The inclusion criteria were as follows: 1) age ≥ 18, 2) primary HCC, and 3) received HCC excision surgery. The patients were excluded if they 1) suffered from multiple metastases, 2) combined with other type of cancers, or 3) the clinical data were missing. Patients with HCC who met the criteria were recruited, and HCC samples were collected in the operation room immediately and stored in −80°C refrigerator. All samples of HCC were confirmed by the pathological diagnosis after surgery.

### RNA-seq of clinical hepatocellular carcinoma samples

The HCC samples were sent for RNA-seq to explore expression of NRGs and functional enrichment. The HCC tissue (2 cm × 2 cm) was immediately put into liquid nitrogen for preservation after excision from the patients. Then, the HCC tissue was ground and lysed in TRIzol reagent (Invitrogen, United States ), and the total RNA was extracted for mRNA sequencing. Concentrations and RNA integrity were verified before the library preparation. Library preparation and sequencing were performed by the Biomarker Technologies Corporation, Beijing, China. Sequencing was performed on a HiSeq2500 instrument (Illumina, United States ) with 150 bp paired-end reads. By calculating risk scores, patients were separated into the high-risk group and the low-risk group. Sequentially, DEGs and functions enrichment were proceeded.

### Statistical analysis

Statistical analyses were completed using IBM SPSS Statistics 23.0 (SPSS Inc., Armonk, NY, United States). Quantitative data are presented as the mean ± SD, and categorical variables are presented as frequency (*n*) or proportion (%). Differences between the two groups were analyzed with an independent samples/paired Student’s *t*-test. Categorical variables were compared using the *χ* ^2^ test with the Yates correction or Fisher’s exact test. Survival curves were created using Kaplan–Meier survival analysis with a log-rank *t*-test. All the statistical tests were two sided with *p* values < 0.05 being considered statistically significant.

## Results

**FIGURE 1:**
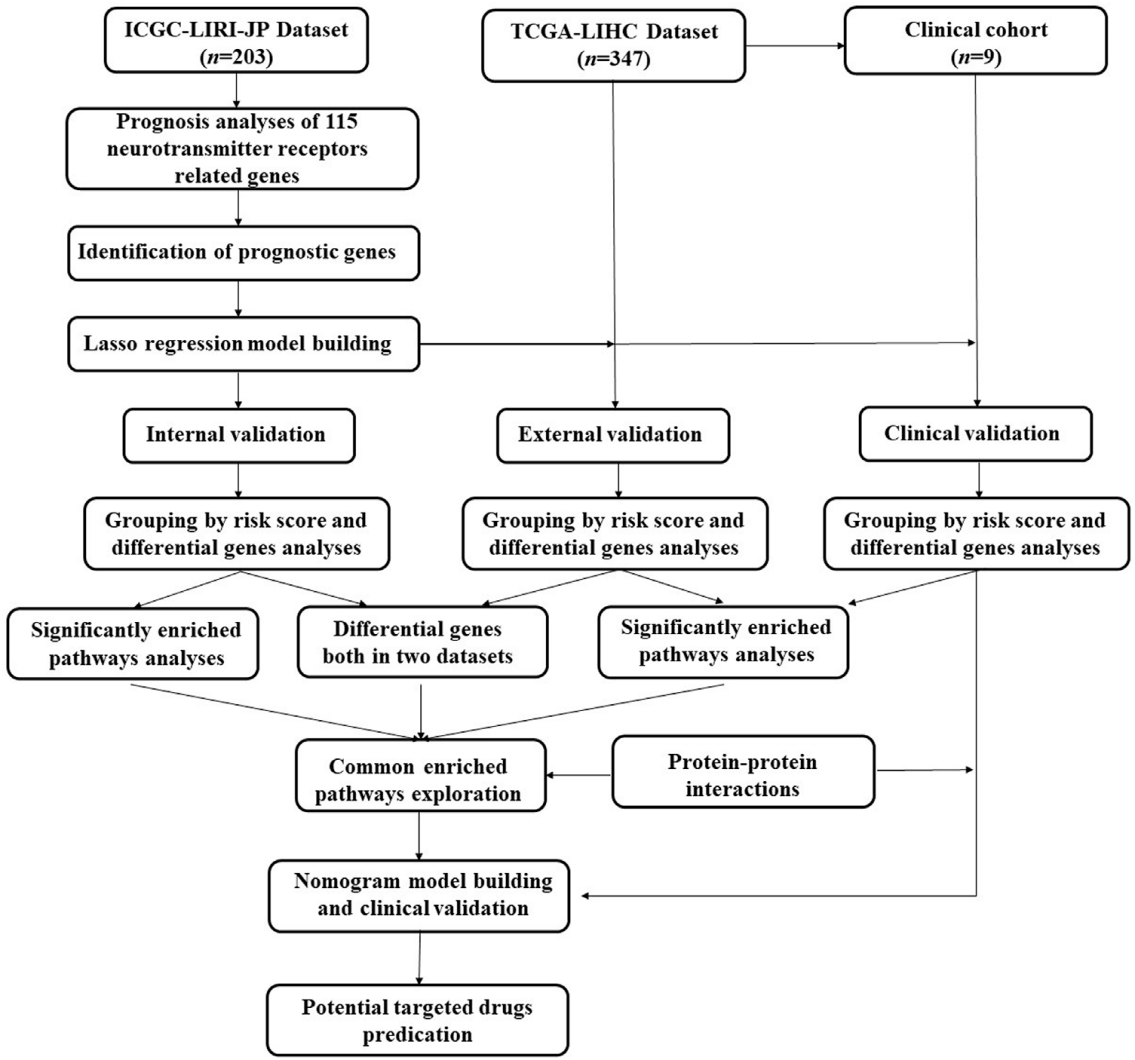
Design flow chart and validation process of prognosis model construction.

The design flow chart and validation process of this study are presented in Figure 1. The full names of the 115 NRGs were provided in the Supplementary Table S1.

### Construction of the prognosis model based on neurotransmitter receptor-related genes in the ICGC-LIRI-JP dataset

**FIGURE 2:**
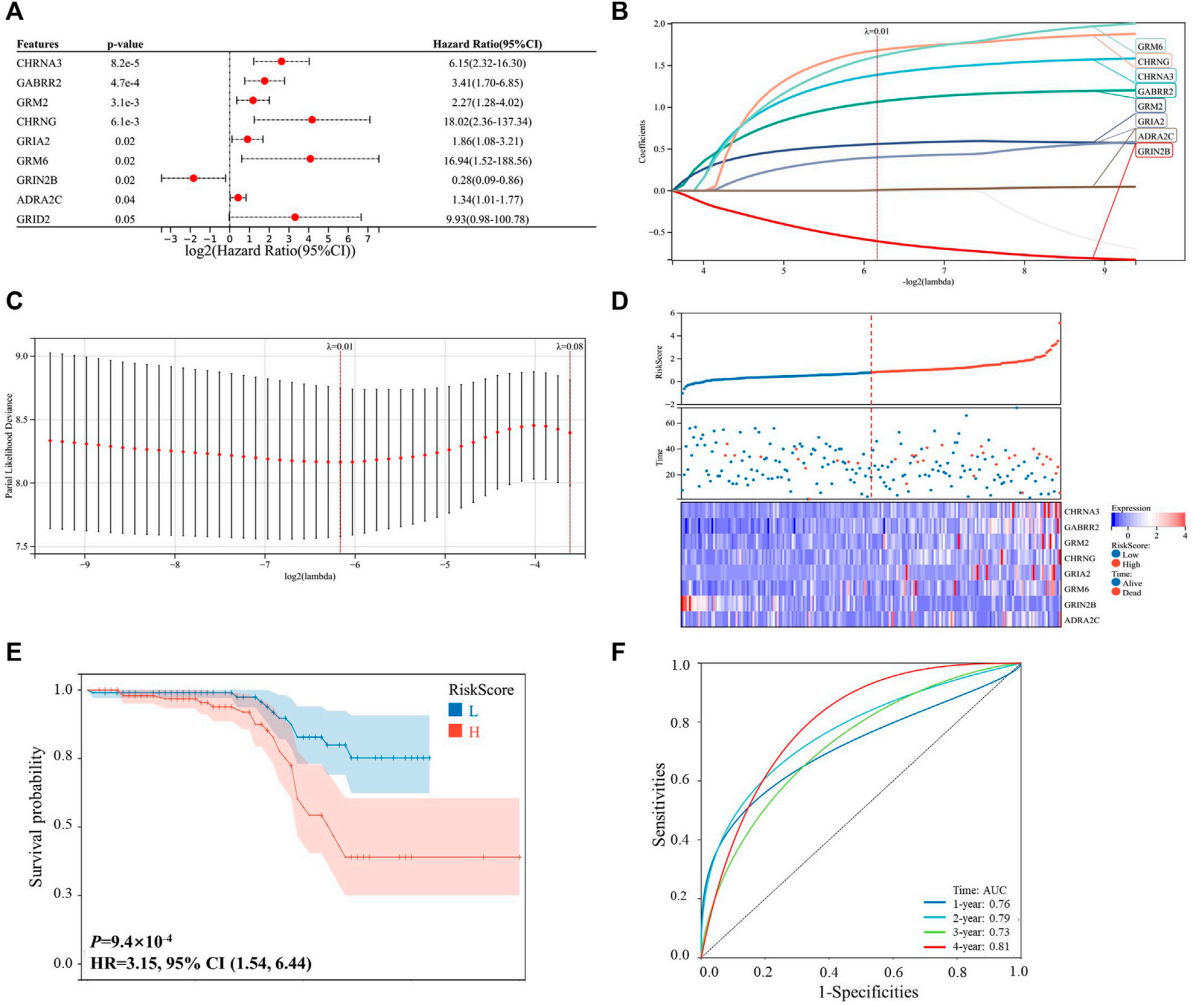
Prognosis construction model based on the neurotransmitter receptor-related genes in the ICGC-LIRI-JP dataset. **(A)** Univariable cox survival analysis of the 115 neurotransmitter receptor-related genes, and nine genes were significantly correlated with the prognosis of patients with HCC. **(B,C)** LASSO regression model construction based on the nine predictive genes in the ICGC-LIRI-JP dataset, and 8 genes were successfully included in the model. **(D)** Distribution of the risk scores, survival status, and expression of the eight critically predictive genes. **(E)** The Kaplan–Meier analysis of overall survival in the high-risk scores group and low-risk scores group. **(F)** The ROC analysis to evaluate the predictive of risk scores efficiency. CI, confidence interval; HCC, hepatocellular carcinoma; ROC, receiver operator characteristic.

As shown in Figure 1, 203 patients with HCC were identified from the ICGC-LIRI-JP dataset, and they were used as the internal validation cohort. By performing the univariable Cox survival analysis of 115 NRGs in the ICGC-LIRI-JP dataset, nine critical genes (CHRNA3, GABRR2, GRM2, CHRNG, GRIA2, GRM6, GRIN2B, ADRA2C, and GRID2), which were significantly correlated with the OS of the patients with HCC, were identified (Figure 2A). Next, these genes were included in the LASSO regression analysis to establish the prognostic model by integrating survival time, survival status, and gene expression data. Finally, 8 genes were successfully included into the model, and the risk score formula is as follows: risk score = 1.389 × CHRNA3 + 1.065 × GABRR2 + 0.560 × GRM2 + 1.683 × CHRNG + 0.400 × GRIA2 + 1.608 × GRM6–0.606 × GRIN2B + 0.006 × ADRA2C (Figures 2B,C). Every patient got a risk score by integrating the expression level of each gene into the formula. The results in Figure 2D showed that patients with higher risk scores displayed more deaths or shorter survival years. Furthermore, the heat map analysis also indicated that patients with high-risk score had generally high expression levels of the eight critically predictive NRGs, except GRIN2B (Figure 2D). Moreover, the survival curve was consistent with the heat map analysis by showing that patients with higher risk score had a worse OS (*p* < 0.001, HR = 3.15, Figure 2E). The subsequent ROC analysis suggested that this risk score model could effectively predict patients with HCC survival, with the area under the curve (AUC) of a 4-year survival reaching to 0.81 (Figure 2F).

### Differentially expressed genes validations and potential pathways enrichments in the ICGC-LIRI-JP dataset

**FIGURE 3:**
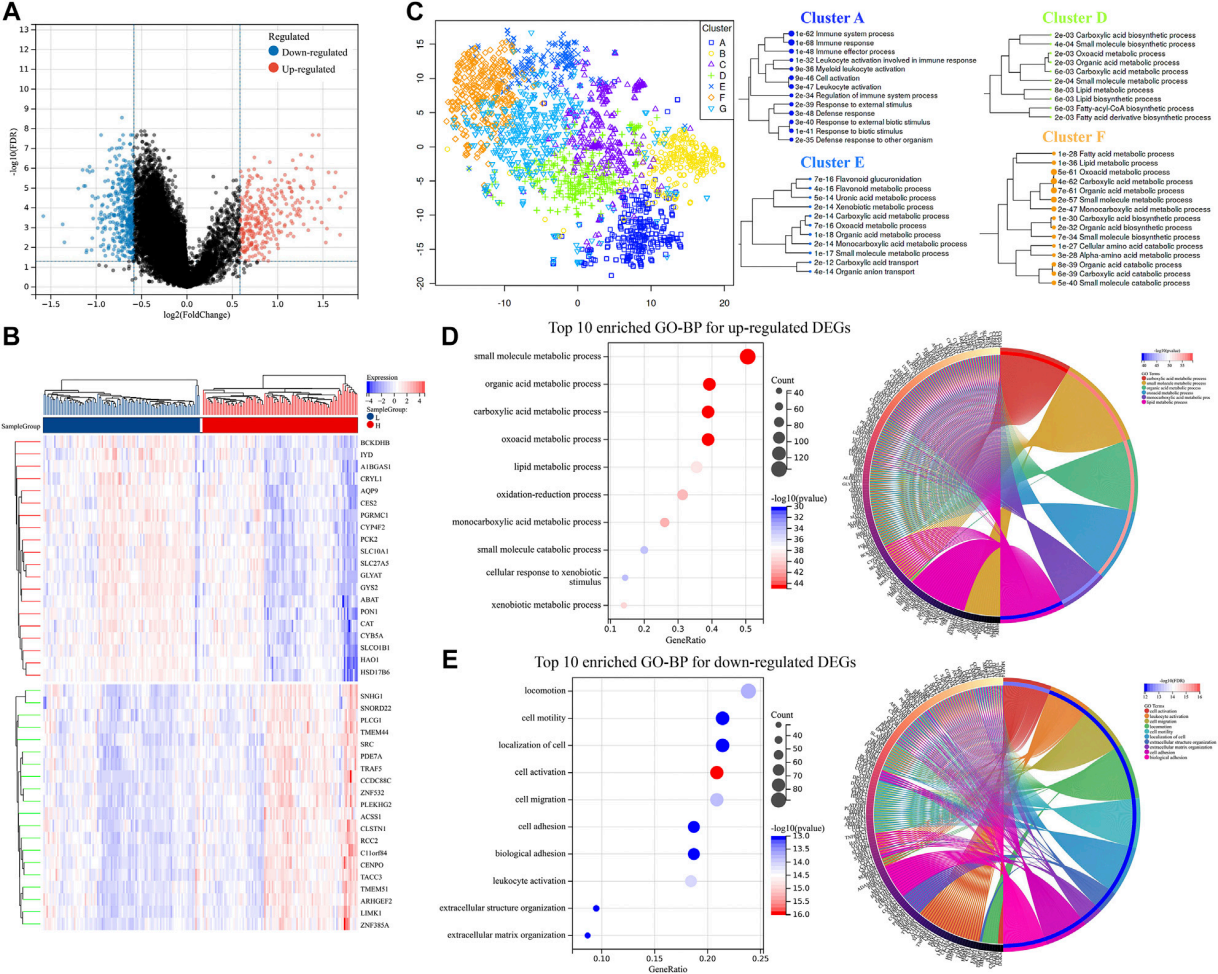
DEGs validation and potential pathway enrichments in the ICGC-LIRI-JP dataset. **(A)** Volcano plot of DEGs. **(B)** DEGs heat map between two groups. **(C)** Map and clusters of DEGs using t-SNE. **(D,E)** Top 10 enriched GO-BPs and circle plots for DEGs in the ICGC-LIRI-JP datasets. DEGs, differentially expressed genes; t-SNE, t-Distributed Stochastic Neighbor Embedding; GO, gene ontology; BP, biological process.

The 203 patients from the ICGC-LIRI-JP dataset were then divided into two groups based on the median of all their risk scores. Furthermore, the DEGs were screened. As shown in Figure 3A; Supplementary Table S2, 412 downregulated DEGs and 290 upregulated DEGs were identified. In addition, the heat map of the top 20 upregulated and downregulated DEGs between the two groups is shown in Figure 3B. Next, by visualizing the DEGs into the different clusters (Figure 3C), it suggested that the DEGs were mainly involved in the immune system process, lipid metabolic process, organic acid metabolic process, oxoacid metabolic process, etc. Subsequently, GO pathway enrichment was performed for upregulated and downregulated DEGs, respectively. The results revealed that metabolic processes, especially organic acid and lipid metabolic processes, cell motility, and leukocyte activation, were enriched (Figures 3D,E; Supplementary Figure S1).

### External validations and potential pathways enrichments in the cancer genome atlas-liver hepatocellular carcinoma dataset

**FIGURE 4:**
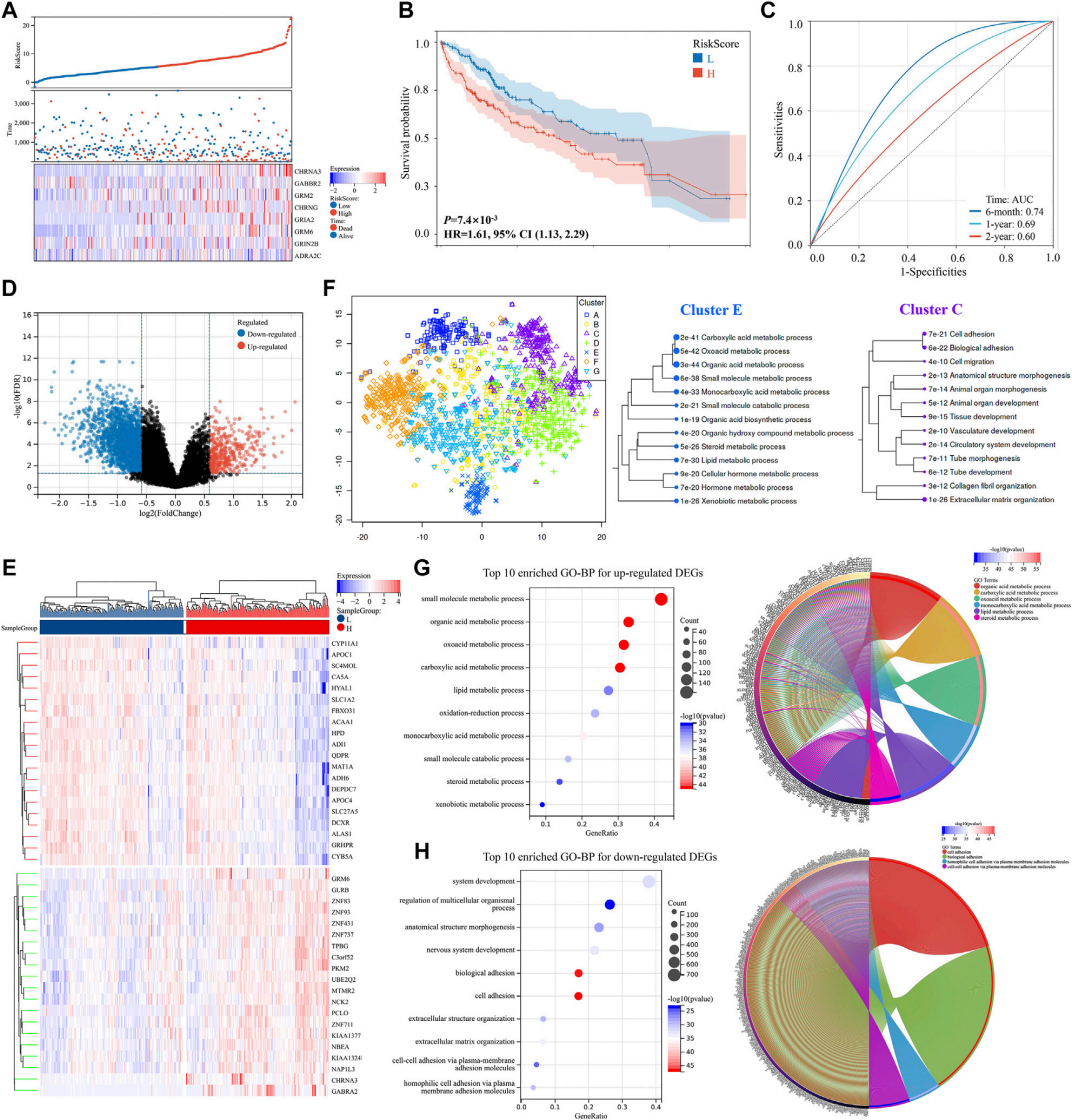
DEGs validation and potential pathway enrichments in the TCGA-LIHC dataset. **(A)** Distribution of the risk scores, survival status, and expression of the eight critically predictive genes. **(B)** The Kaplan–Meier analysis of overall survival in the high-risk scores group and low-risk scores group. **(C)** The ROC analysis to evaluate the predictive efficiency of risk scores. **(D)** Volcano plot of DEGs. **(E)** Heat map of DEGs between two groups. **(F)** Map and clusters of DEGs using t-SNE. **(G,H)** Top 10 enriched GO-BP and circle plots for DEGs in the TCGA-LIHC datasets. DEGs, differentially expressed genes; t-SNE, t-Distributed Stochastic Neighbor Embedding; GO, gene ontology; BP, biological process. ROC, receiver operator characteristic.

The TCGA-LIHC dataset was used for the external validation to validate the risk score model’s effectiveness. Three hundred forty seven patients with HCC were identified from the TCGA-LIHC dataset, and each of them got a score calculated with the risk score formula. They were then separated into two groups based on the median of all their risk scores. As shown in Figures 4A,B, patients from the high-risk score group had a worse prognosis compared to those from the low-risk score group (*p* < 0.001, HR = 1.61). Additionally, the prognostic model exhibited a promising predictive capability, and the AUC of a 6 months survival reached to 0.74 (Figure 4C).

A volcano plot of DEGs showed that 2,320 genes were downregulated and 445 genes were upregulated between the two groups (Figure 4D; Supplementary Table S3). Moreover, the heat map of the top 20 upregulated and downregulated DEGs, respectively, was shown in Figure 4E. DEGs visualization and GO enrichment analysis revealed that similar pathways were enriched compared to the ICGC-LIRI-JP dataset (Figures 4F–H and Supplementary Figure S2), indicating that NRGs might play key roles in acid substances and lipid metabolic processes, cell adhesion, and migration in HCC.

### Mutual differentially expressed genes validation and pathways enrichment

**FIGURE 5:**
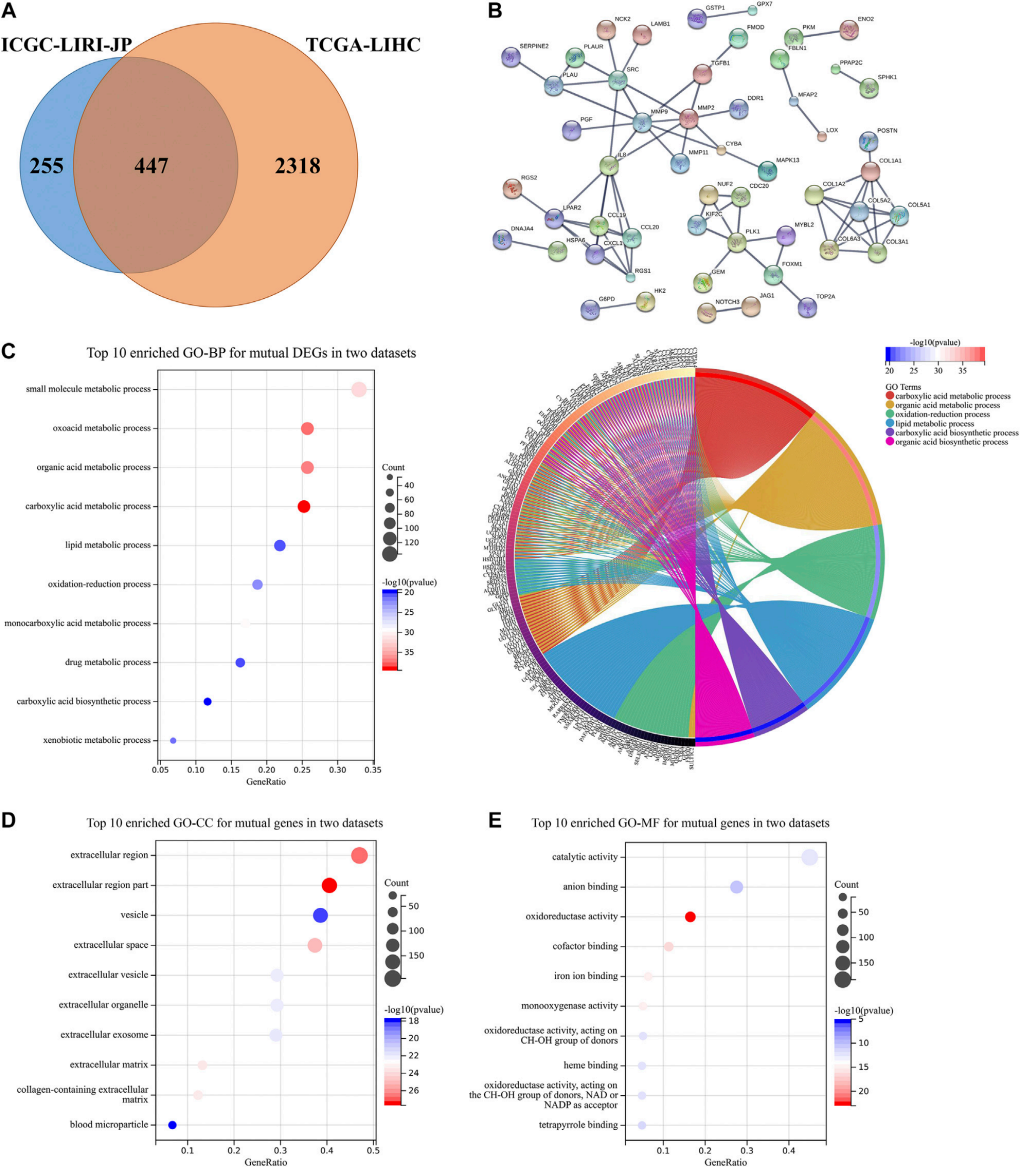
DEGs validation and potential pathway enrichments both in the TCGA-LIHC dataset and the ICGC-LIRI-JP dataset. **(A)** Venn diagram was constructed to show DEGs both in the TCGA-LIHC dataset and the ICGC-LIRI-JP dataset. **(B)** PPI construction of DEGs both in two datasets. **(C)** Top 10 enriched GO-BP and circle plots for mutual DEGs. **(D,E)** Top 10 enriched GO-CC and GO-MF for mutual DEGs. DEGs, differentially expressed genes; GO, gene ontology; BP, biological process; CC, cellular component; MF, molecular function; PPI, protein-protein interaction.

A total of 447 mutual DEGs which were both in the ICGC-LIRI-JP dataset and the TCGA-LIHC dataset were screened (Figure 5A). The PPI network revealed that complex connections among mutual DEGs, with some critical proteins accounting for tumor progression [i.e., Src, matrix metalloproteinase (MMP) family, and chemokine family], are being identified (Figure 5B) (Kim et al., 2009; Kessenbrock et al., 2010). GO enrichment analysis also suggested that mutual DEGs mainly participate in acid substance and lipid metabolic processes, which is consistent with the above results in Figures 3, 4 (Figures 5C–E).

### Risk score model validation, differentially expressed genes screen and potential pathways enrichments in the clinical cohort

**FIGURE 6:**
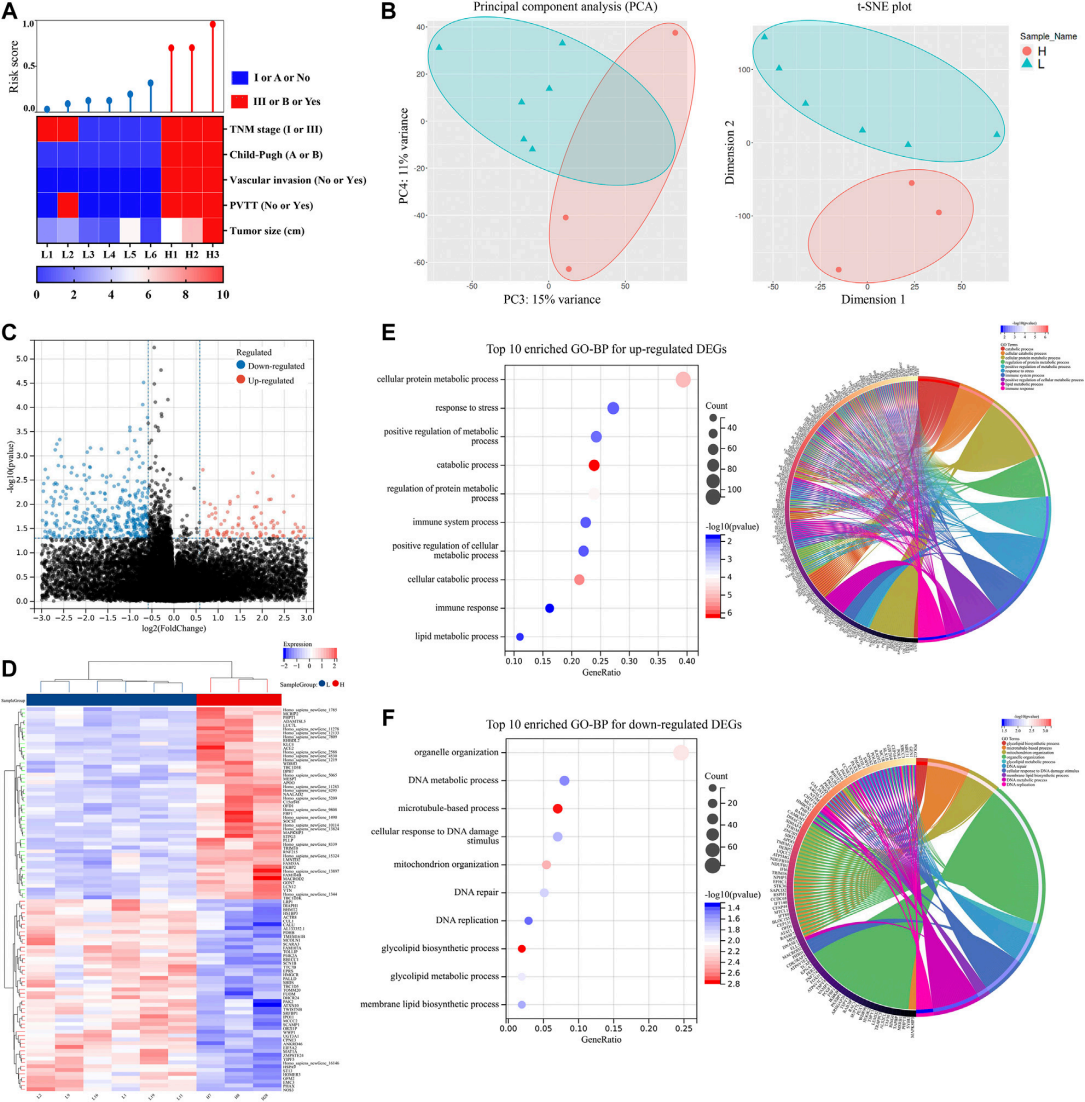
Risk score model validation, DEGs screen and potential pathways enrichments using nine HCC samples. **(A)** Correlations among risk score and clinical characteristics. **(B)** PCA and t-SNE plot of nine HCC samples. **(C)** Volcano plot of DEGs. **(D)** Heat map of DEGs between two groups. **(E)** Top ten enriched GO-BP and circle plots for up-regulated DEGs. **(F)** Top ten enriched GO-BP and circle plots for downregulated DEGs. DEGs, differentially expressed genes; GO, gene ontology; BP, biological process; HCC, hepatocellular carcinoma; PCA, principal component analysis; t-SNE, t-distributed Stochastic Neighbor Embedding; TNM, clinicopathological stage; PVTT, portal vein tumor thrombus.

We performed a clinical trial to further validate the model’s effectiveness. From April 2021 to May 2021, nine patients with HCC who met the criteria were recruited, and the collected HCC samples were sent for RNA-seq. The dataset for RNA-seq was supplied as the Supplementary Material. By calculating the risk score, patients were separated into two groups. The clinical characteristics of these patients are shown in Table 1 and Figure 6A. Patients in the high-risk score group exhibited more advanced tumor stage and Child-Pugh stage, larger tumor size, more vascular invasion, and portal vein tumor thrombus (PVTT). PCA and t-SNE analyses also suggested that the gene distribution pattern was significantly different in patients of the two groups (Figure 6B). Nine hundred fifteen DEGs were identified between the two groups and were shown in the volcano plot (Figure 6C; Supplementary Table S4). Furthermore, the heat map of the top 20 upregulated and downregulated DEGs was shown in Figure 6D, respectively. In signaling pathway prediction, top-enriched GO pathways were mainly involved in the cellular metabolic process, response to stress, and immune-related processes, which were consistent with the results both in the ICGC-LIRI-JP dataset and the TCGA-LIHC dataset. Organelle organization, DNA-related pathways, and glycolipid metabolic processes were enriched as well (Figures 6E,F). These results support the above hypothesis that NRGs may play critical roles in cellular metabolic processes (especially organic acid, inorganic acid, and lipid metabolism) and in immune response in HCC.

**TABLE 1:** Clinical characteristics of HCC patients between two groups divided by risk score.

	Low-risk score group (*n* = 6)	High-risk score group (*n* = 3)	*p* value
Risk score	0.1 (0.0)	0.8 (0.1)	0.00
Gender (male/female)	6/0	3/0	1.00
Age (year)	52.3 (5.5)	58.3 (4.1)	0.50
Height (cm)	172.5 (1.7)	169.3 (4.7)	0.45
Weight (kg)	70.5 (3.1)	70.2 (5.1)	0.95
ASA stage (I/II)	3/3	0/3	0.46
Child-Pugh stage (I/II)	6/0	0/3	0.01
TNM stage (I/III)	4/2	0/3	0.17
Hypertension (Yes/No)	4/2	3/0	0.50
Hepatitis (Yes/No)	6/0	3/0	1.00
Cirrhosis (Yes/No)	6/0	3/0	1.00
PVTT (Yes/No)	2/4	3/0	0.17
Artery invasion (Yes/No)	0/6	3/0	0.01
Primary HCC (Yes/No)	6/0	3/0	1.00
Tumor number (Single/Multiple)	6/0	2/1	0.33
Tumor size	2.8 (0.6)	9.0 (1.3)	0.00
ALT (U/L)	56.8 (25.7)	23.0 (5.5)	0.40
AST (U/L)	45.5 (14.4)	24.7 (8.2)	0.37
TBiL (mmol/L)	14.9 (2.1)	14.5 (0.9)	0.89
ALB (g/L)	43.5 (0.9)	41.1 (2.5)	0.30
AFP (ng/ml)	56.5 (38.8)	139.2 (134.9)	0.61
INR	1.0 (0.0)	1.1 (0.1)	0.16
Cr (μmol/L)	56.2 (5.1)	80.7 (4.3)	0.02
White blood cell (10^9^/L)	5.6 (0.7)	5.4 (0.5)	0.89
HB (g/L)	132.0 (7.9)	146.0 (6.4)	0.29
PLT (10^9^/L)	214.5 (50.8)	146.0 (15.3)	0.39

Variables are shown as “mean (SD)”. HCC, Hepatocellular carcinoma; ASA, American Society of Anesthesiologists; TNM, Clinicopathological stage; PVTT, portal vein tumor thrombus; ALT, Alanine transaminase; AST, aspartate aminotransferase; TBIL, total bilirubin; ALB, serum albumin; AFP, alpha-fetoprotein; INR, International Normalized Ratio; Cr, creatinine; HB, hemoglobin; PLT, platelets; SD, standard deviation.

The PPI network was constructed to show the relationship of DEGs. As shown in Supplementary Figure S3, several critical proteins in tumor development and progression, such as PTEN, MAPK8, CUL1, and RAC3, were enriched.

### Nomogram construction and potential targeted drugs prediction for hepatocellular carcinoma

**FIGURE 7:**
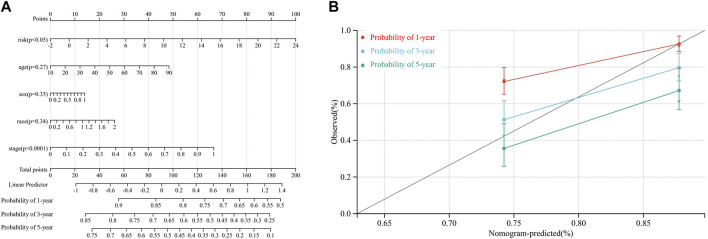
. Nomogram to predict the probability of a 1-year, a 3-year, and a 5-year OS in patients with HCC based on the TCGA-LIHC dataset. **(A)** the nomogram was constructed based on five clinical factors, and results suggested that the risk score and TNM stage significantly affected the OS of patients with HCC. For the factor sex, 0 represents male and 1 represents female; for the factor race, 0 represents the white, 1 represents the Asian, and 2 represents others; for tumor stage, 0 represents stage I or II while 1 represents stages III and IV **(B)**. Calibration plots of the nomogram for a 1-year, a 3-year, and a 5-year OS.

By integrating risk score, age, sex, race, and TNM stage, we developed a novel prognostic nomogram to establish a reliable and quantifiable method for predicting patients with HCC survival. According to Figure 7A, risk score and TNM stage significantly affected the OS of patients with HCC (both *p* < 0.05). In addition, the calibration plots of the nomogram also suggested a reliable prediction effect based on the model (Figure 7B).

**FIGURE 8:**
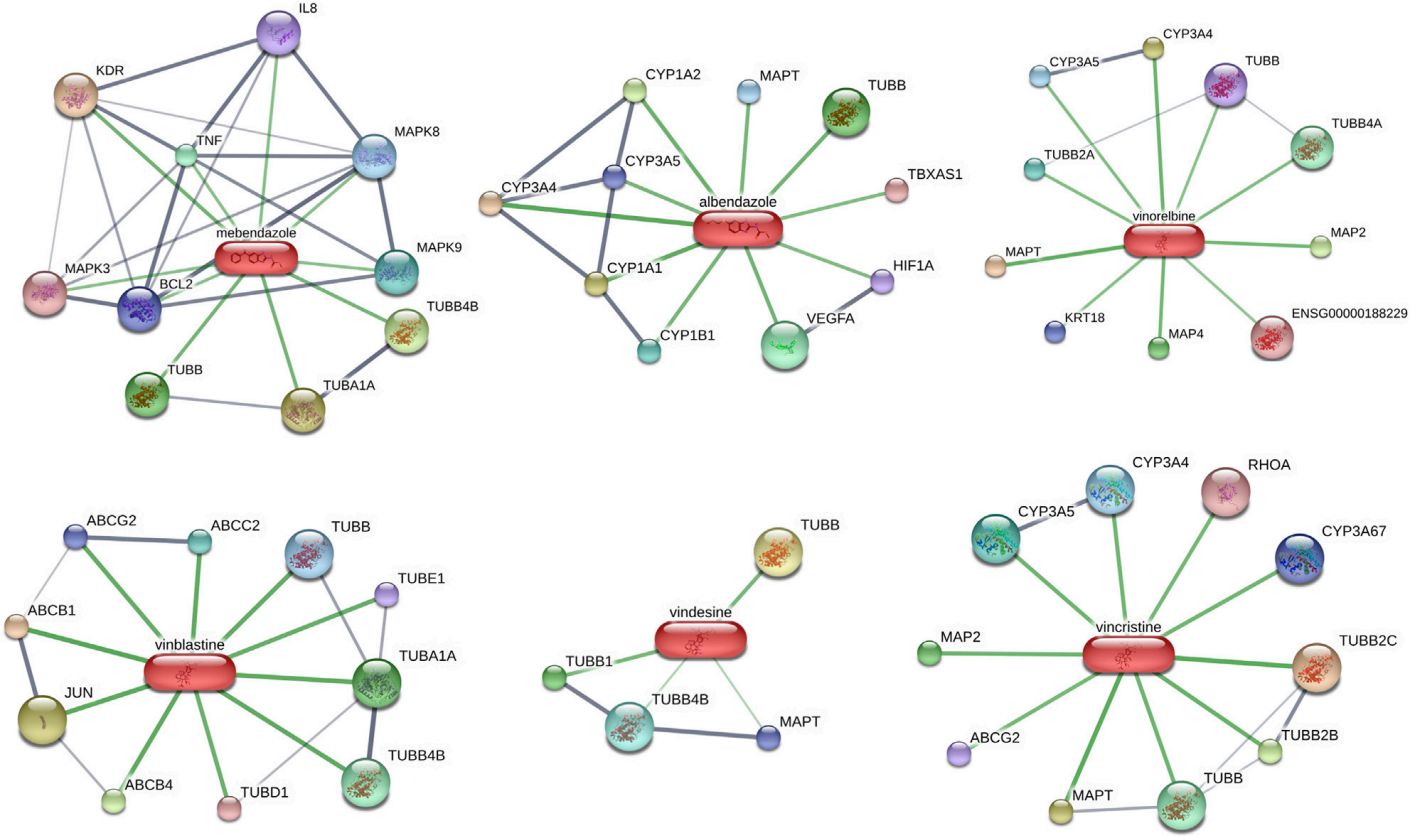
Potential targeted drugs prediction and interactions between top six drug candidates and proteins based on DEGs of nine HCC samples.

Potential targeted drugs for HCC treatment were predicted by using the CMAP dataset. The DEGs of the clinical HCC samples were put into the dataset, and the top six candidate drugs which could reverse changes in DEGs were shown in Table 2. Interestingly, all six candidate drugs were tubulin inhibitors, and studies have revealed the importance of tubulin in tumor progression (Gudimchuk and McIntosh, 2021). Two of them were benzimidazole-related, which were mainly used for worm infections. Others were vinca-alkaloid-related, which has been widely used for tumor treatment (Jordan and Wilson, 2004). In addition, interactions between the top 6 drug candidates and proteins were presented in Figure 8. These results suggest that benzimidazole-related drugs and vinca-alkaloid-related drugs may be potential targeted drugs for patients with HCC with high-risk scores.

**TABLE 2:** Top 6 small molecular drug candidates for HCC treatments.

Name	Cell line	Major introduction	Major function	Correlation score
Mebendazole	Huh7	Mebendazole is a synthetic benzimidazole derivate and anthelmintic agent. It is used commonly for parasitic worm infections.	Tubulin inhibitor	−0.57
Albendazole	Huh7	Albendazole is a benzimidazole medication used for the treatment of a variety of parasitic worm infestations.	Tubulin inhibitor	−0.52
Vinorelbine	Huh7	Vinorelbine is a semisynthetic vinca alkaloid. Vinorelbine binds to tubulin and prevents formation of the mitotic spindle, resulting in the arrest of tumor cell growth in metaphase.	Tubulin inhibitor	−0.52
Vinblastine	HepG2	Antitumor alkaloid isolated from Vinca rosea. It binds to tubulin and inhibits microtubule formation, resulting in disruption of mitotic spindle assembly and arrest of tumor cells in the M phase of the cell cycle.	Tubulin inhibitor	−0.52
Vindesine	Huh7	Vindesine is an anti-mitotic vinca alkaloid used in chemotherapy. It is used to treat many different types of cancer.	Tubulin inhibitor	−0.50
Vincristine	Huh7	Vincristine is a vinca alkaloid and used as a chemotherapy drug. It has a role as a tubulin modulator, a microtubule-destabilizing agent.	Tubulin inhibitor	−0.46

## Discussion

The nervous system-cancer crosstalk has emerged as a novel and crucial cancer progression facilitator with the recent increasing attention to the indispensable roles of systemic regulation and tumor (Jiang et al., 2020a; Zahalka and Frenette, 2020; Huang et al., 2022). Studies have revealed that the nervous system regulates development, metastasis, tumor microenvironment, and vascular formation of tumors (Jiang et al., 2020b; Zahalka and Frenette, 2020). However, as an emerging area of cancer science, the interactions and crosstalk between the nervous system and cancer remain to be explored.

As a key component of the nervous system, neurotransmitters and neurotransmitter receptors participate in the information transmission between neurons and other types of cells, including tumor cells (Jiang et al., 2020a; Hodo et al., 2020; Gysler and Drapkin, 2021). Reports also suggest that neurotransmitter receptors are widely expressed in cancer cells and contribute to tumor proliferation, tumor angiogenesis, metastasis, and tumor microenvironment regulation, including HCC (Jiang et al., 2020a; Hodo et al., 2020). Nevertheless, the comprehensive effects of the different types of neurotransmitter receptors on HCC progression were still unclear. Therefore, we constructed a novel prognostic model based on 115 NRGs and explored potential mechanisms that NRGs may be involved in. In our study, we systematically analyzed the effects of NRGs on the prognosis of patients with HCC. Furthermore, eight critical genes were selected for model construction. Next, we further verified the model’s effectiveness in internal validation, external validation, and clinical validation. The survival analysis revealed that patients with a high-risk score generally had a worse prognosis. All validations showed good consistency and availability, which indicated that our prognostic model was practical.

The DEGs were screened by dividing patients into two groups based on risk score. Functional enrichments suggest that NRGs may play critical roles in cellular metabolic processes (especially organic acid, inorganic acid, and lipid metabolism), immune response, and cell motility. Studies have also validated the lipid metabolism’s prominent status in cancer progression. Approaches to target dysfunctional lipid metabolism show prospects (Bian et al., 2021). Recent studies also have mentioned the regulation among NRGs, immune response, and cellular metabolism (Jensen et al., 2021; Mohammadpour et al., 2021; Wan et al., 2021). Apart from that, drug prediction revealed that benzimidazole-related and vinca-alkaloid-related drugs could effectively reverse these changes. These findings bring a new view to cancer neuroscience research and therapeutic directions for patients with HCC with high-risk scores.

Among eight critical NRGs, four genes (GRM2, GRM6, GRIA2, and GRIN2B) are derived from the glutamate receptor family, which suggests that glutamate receptor family play important roles in HCC development. Glutamine is an indispensable nutrient for cell proliferation and nucleotide biosynthesis and can also produce energy via the tricarboxylic acid cycle (Prickett and Samuels, 2012; Stepulak et al., 2014). In addition, glutamine also serves as a substrate for fatty acid synthesis in hypoxic cells or cells with HIF-1 activation (Yi et al., 2019). Studies have also suggested that glutamine plays a key role in cancer metabolism. Moreover, glutamatergic signaling pathway dysfunction was observed in multiple types of cancers (Stepulak et al., 2014). The abnormal activation of the glutamatergic signaling pathway was also correlated with tumor growth, tumor angiogenesis, and tumor metastasis (Venkataramani et al., 2019; Yi et al., 2019). A study by Zeng et al. (2019) found that, in a breast-to-brain metastasis model, N-methyl-D-aspartate receptor (NMDARs) activation promoted metastatic colonization of breast cancer cells to the brain. Then, formation of pseudo-tripartite synapses between cancer cells and glutamatergic neurons was observed. In gastric cancer, Xu et al. (2018) revealed that the GRINA expression in cancer was significantly higher than that in normal tissues, GRINA also promoted the proliferation, migration, and invasion capacity of gastric cancer cells. There are reports that also identified the critical glutamate metabotropic receptors’ effects in antitumor immunity (Kansara et al., 2019; Wan et al., 2021). For instance, Wan et al. (2021) showed that GRM4 played an important role in negatively modulating antitumor immunity, and global GRM4 knockout or pharmacological inhibition of GRM4 led to a significant inhibition of tumor growth in multiple tumor models. However, research on GRM2 and GRM6 is lacking, which merits further investigation. As the only protective factor among eight genes, high GRIN2B expression is associated with a low-risk score and a better prognosis for patients with HCC. Several studies also verified this hypothesis (Chung et al., 2011; Park et al., 2011). Chung et al. (2011) found that GRIN2B hypermethylation was more frequent in invasive pulmonary adenocarcinoma, which indicated that GRIN2B dysfunction might facilitate tumor invasion. Park et al. (2011) showed that the promoter CpG island methylation of GRIN2B changed significantly during breast cancer progression. Therefore, the effects of the glutamate receptor family on cancer were complex and needed comprehensive consideration.

The other four genes are derived from the acetylcholine receptor family (CHRNG and CHRNA3), the GABAergic receptor family (GABRR2), and the adrenergic receptor family (ADRA2C). Studies have also revealed connections between acetylcholine receptors and multiple types of cancers such as lung cancer, head and neck cancer, and gastric cancer (Hayakawa et al., 2017; Silva et al., 2019; Yi et al., 2021). Also, an increasing number of studies have reported the key functions of GABAergic receptors and adrenergic receptors in tumor development (Zahalka et al., 2017; Jiang et al., 2019; Iftikhar et al., 2021). However, studies of these four crucial genes in HCC are still scarce, which needs more exploration.

Many prognostic models have been developed for HCC, such as the TNM stage, the Barcelona Clinic Liver Cancer (BCLC) system, the Cancer of the Liver Italian (CLIP) Program, and the Japan Integrated Staging (JIS) score (Author anonymous, 2000; Kudo et al., 2003; Reig et al., 2021), for better prognosis prediction and supply of individual treatment and follow-up plans. These models mainly select clinical factors, including tumor size, metastasis condition, vascular invasion, etc. With the development of the Human Genome Project and RNA-seq technology, it has become easy for doctors to acquire more information at the gene level (Uhlen et al., 2017). Therefore, new prognostic models, based on gene expression, showed good prospects. Previous studies have suggested a good predictive value of new models based on specific gene clusters such as epithelial-mesenchymal transition-related genes, pyroptosis-related genes, and ferroptosis-related genes (Fu and Song, 2021; Wu et al., 2021; Wan et al., 2022). In this study, we constructed a novel prognostic model based on NRGs, and it showed a good predictive value both in public HCC datasets and in clinical validation.

Some limitations of the present study are worth noting. Firstly, functional enrichment validation by fundamental experiments is needed in the next step. Secondly, gene function and effects of crucial NRGs should be identified by *in vitro* study. Thirdly, a multiple-center, large sample size, and long-term follow-up clinical trial are needed to validate and improve our prognostic model.

## Conclusion

Our study demonstrated that NRGs correlated tightly with the development of HCC. Furthermore, NRGs are promising targets for HCC treatment and prognostic prediction. We also successfully constructed a prognostic model based on critical NRGs and further tested the internal, external, and clinical validation effectiveness. We found that the cellular metabolic processes (especially the acid substances and lipid metabolism) and immune response were significantly enriched using functional analysis. Potential targeted drugs prediction suggests that benzimidazole-related and vinca-alkaloid-related drugs may be potential targeted drugs for patients with HCC with high-risk scores. These findings may provide new targets and translational applications for clinical HCC therapy.

## Data Availability

The datasets presented in this study can be found in online repositories. The names of the repository/repositories and accession number(s) can be found in the article/Supplementary Material.

## Abbreviations

Ach, acetylcholine; ASA, American Society of Anesthesiologists; ALB, serum albumin; AFP, alpha-fetoprotein; ALT, Alanine transaminase; AST, aspartate aminotransferase; AUC, area under the curve; DEGs, differentially expressed genes; E, epinephrine; HCC, hepatocellular carcinoma; NRGs, neurotransmitter receptor-related genes; LASSO, least absolute shrinkage and selection operator; GO, gene ontology; ROC, receiver operator characteristic curve; OS, overall survival; GABA, gamma-aminobutyric acid; NE, norepinephrine; ICGC, International Cancer Genome Consortium; PCA, principal component analysis; t-SNE, t-Distributed Stochastic Neighbor Embedding; PPI, protein-protein interaction; TNM, Clinicopathological stage; PVTT, portal vein tumor thrombus; TBIL, total bilirubin; INR, International Normalized Ratio; Cr, creatinine; HB, hemoglobin; PLT, platelets; SD, standard deviation.
