# Supplementary figures and images for: Comprehensive analysis to identify the neurotransmitter receptor-related genes as prognostic and therapeutic biomarkers in hepatocellular carcinoma
Source: Front Cell Dev Biol. 2022 Aug 5;10:887076. doi: 10.3389/fcell.2022.887076 (PMC9388745; doi:10.3389/fcell.2022.887076)

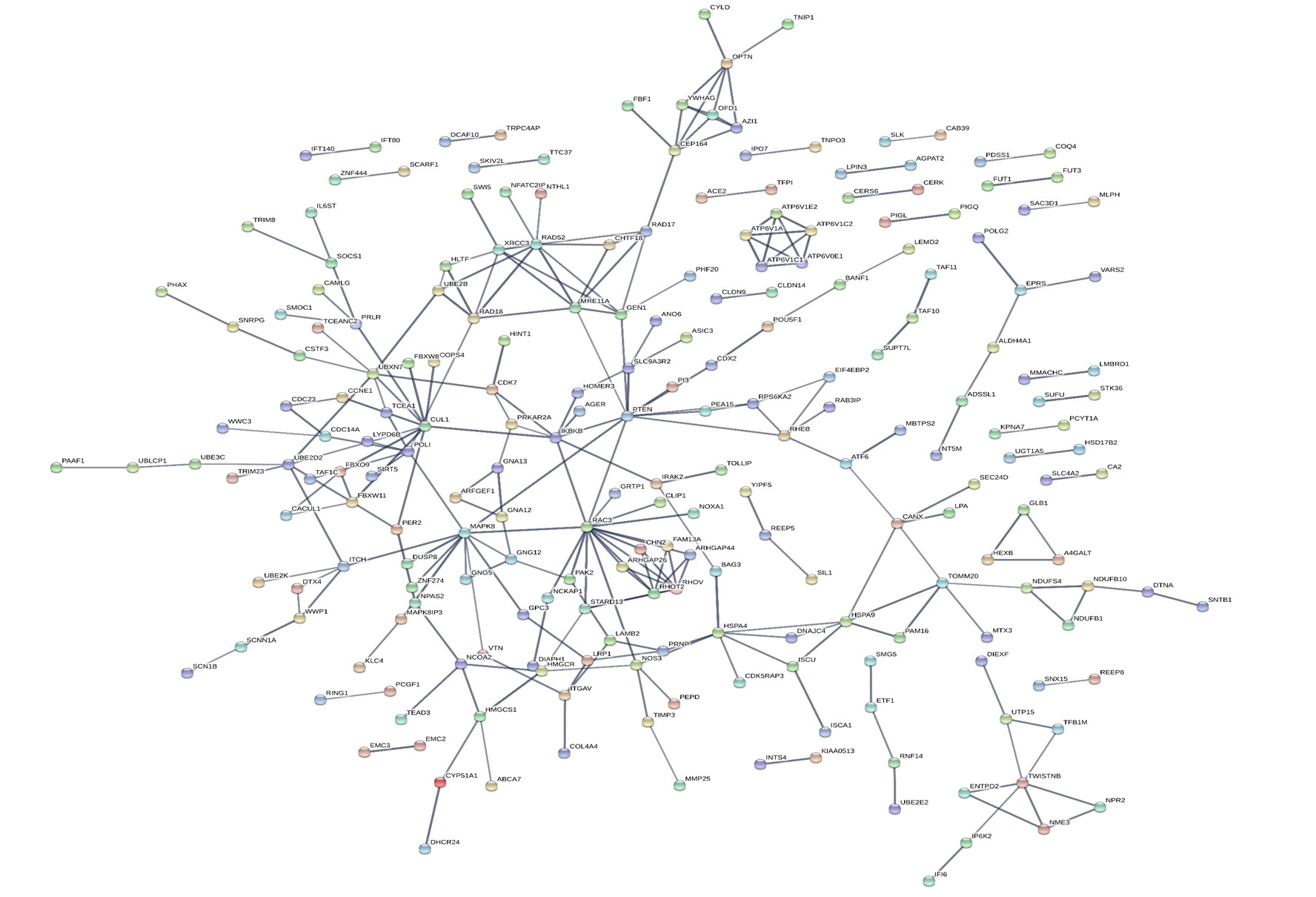

Supplement: Supplementary file 2 [file Image3.JPEG]

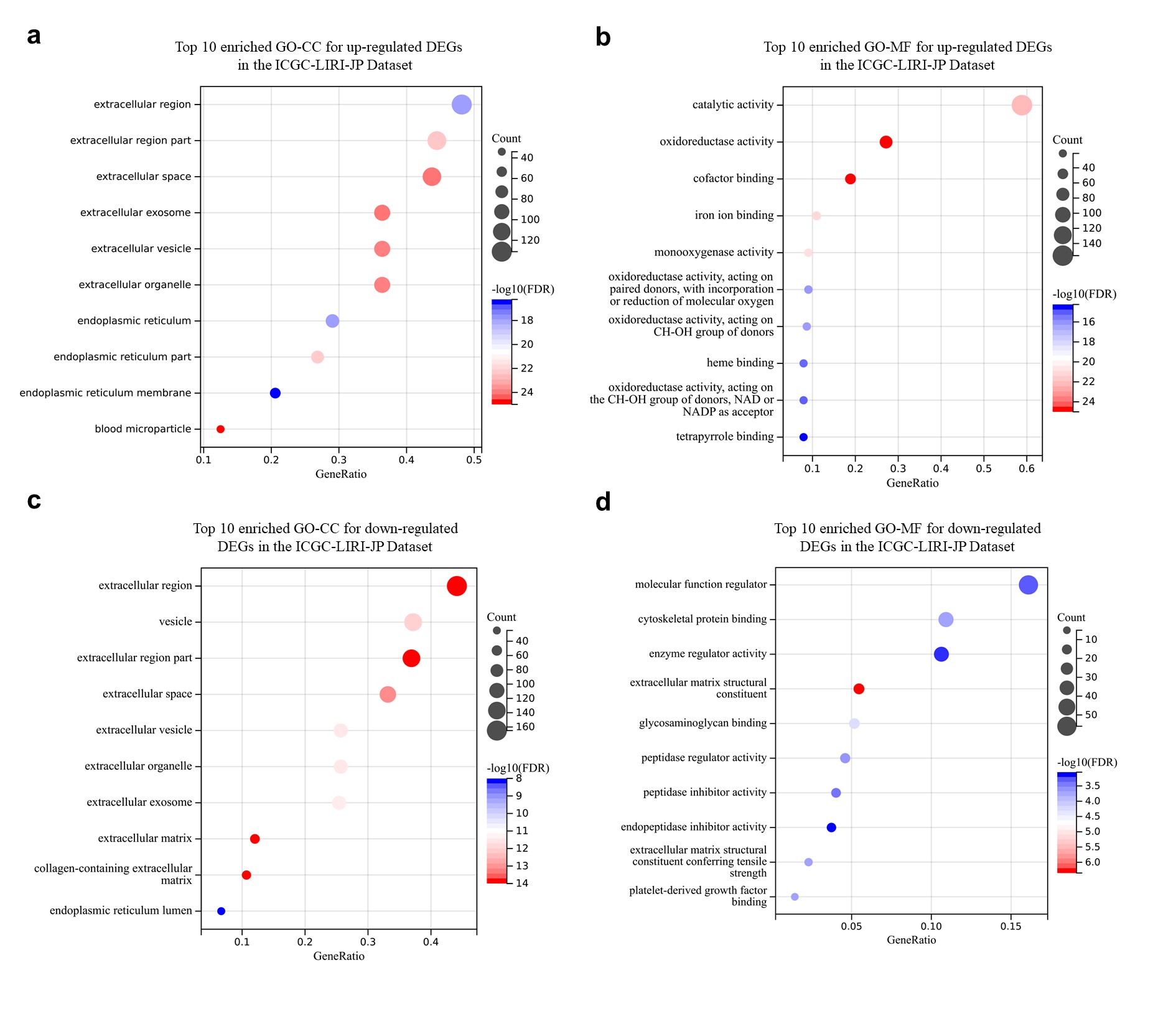

Supplement: Supplementary file 5 [file Image1.JPEG]

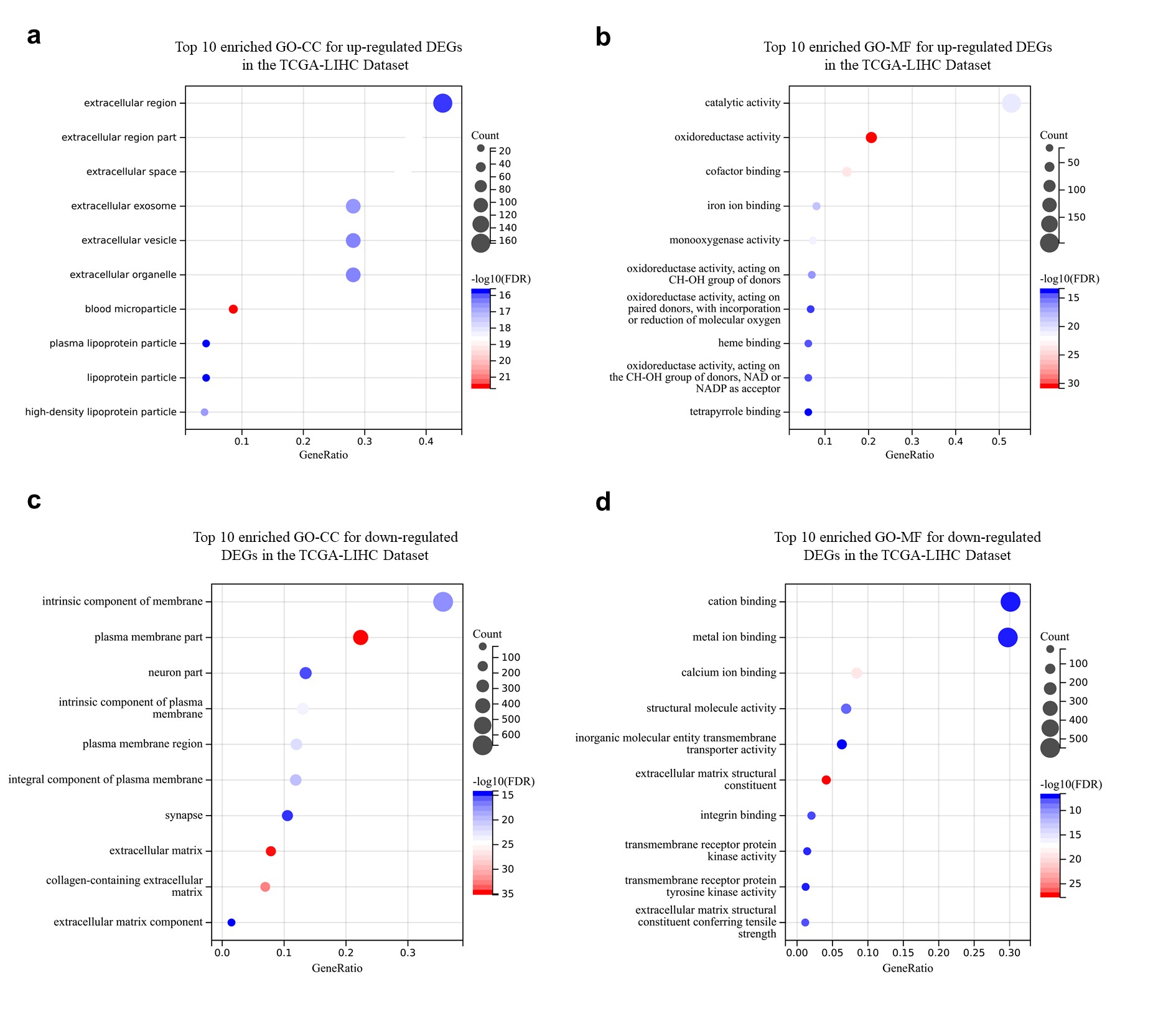

Supplement: Supplementary file 6 [file Image2.JPEG]
